# Associations between various anthropometric indices and hypertension and hyperlipidaemia: a cross-sectional study in China

**DOI:** 10.1186/s12889-024-20505-w

**Published:** 2024-11-04

**Authors:** Chuyao Feng, Cihang Lu, Kang Chen, Bo Song, Zhongyan Shan, Weiping Teng

**Affiliations:** 1 Department of Endocrinology and Metabolism, Institute of Endocrinology, National Health Commission Key Laboratory of Diagnosis and Treatment of Thyroid Diseases, The First Hospital of China Medical University, Liaoning Province, Shenyang, 110001 People’s Republic of China; 2 Department of Ophthalmology, The First Hospital of China Medical University, Liaoning Province, Shenyang, 110001 People’s Republic of China

**Keywords:** Anthropometric indices, Hypertension, Hyperlipidaemia, Waist-to-height ratio, Body roundness index, Body mass index

## Abstract

**Background:**

This study aims to explore the association and determine the distinguished potential of anthropometric adiposity indices in screening for hypertension and hyperlipidaemia in the Chinese population.

**Methods:**

A recent nationwide cross-sectional study, called the Thyroid Disorders, Iodine State, and Diabetes Epidemiological Survey (TIDE 2015–2017), provided the newest data on the relationships between anthropometric adiposity indices and hypertension and hyperlipidaemia and included 65,231 subjects. The area under the curve (AUC) was used to assess the feasibility of using these indices to distinguish hypertension and hyperlipidaemia. After age stratification, a restricted cubic spline (RCS) fitted for generalized linear regression was used to visualize the relationships of the body mass index (BMI), waist circumference (WC), the waist-to-height ratio (WHtR), the body roundness index (BRI), and the “a body shape index” (ABSI) with hypertension and hyperlipidaemia.

**Results:**

The results showed that there were significant differences in the BMI, WC, the WHtR, the BRI, and the ABSI among the different age groups (*P* < 0.0001). After adjusting for sex, age, education, income, smoking status, urban or rural residence, and ethnicity in model, The WHtR and BRI had greater discriminatory power in identifying hypertension (AUC = 0.665, 95% confidence interval (CI) 0.660–0.671 for both), hypercholesterolaemia (AUC = 0.629, 95% CI 0.624–0.634 for both), and high low-density lipoprotein cholesterol (LDL-C; AUC = 0.659, 95% CI 0.653–0.664 for both) status in the overall population. When distinguishing hypertriglyceridaemia among the general population, the BMI (AUC = 0.711, 95% CI 0.706–0.716) and WC (AUC = 0.715, 95% CI 0.710–0.720) had greater discriminatory ability than the other anthropometric indices did. The BMI (AUC = 0.631, 95% CI 0.625–0.637) had the highest power for low high-density lipoprotein cholesterol (HDL-C) status in the general population.

**Conclusions:**

Several anthropometric indices show significant correlation with hypertension and hyperlipidaemia. The WHtR and BRI were better in distinguishing hypertension, hypercholesterolaemia and high LDL-C status, while the BMI was better in hypertriglyceridaemia and low HDL-C status. The use of combined indices, such as the BMI, WC, the WHtR and the BRI, can be included in an individual’s medical history and can be used as tools for cardiovascular health screening, which may yield superior results for public health.

**Supplementary Information:**

The online version contains supplementary material available at 10.1186/s12889-024-20505-w.

## Introduction

Hypertension is an increasingly serious global public health problem and the diagnosis in Chinese adults is advised at a systolic blood pressure (SBP) ≥ 140 mmHg and/or a diastolic blood pressure (DBP) ≥ 90 mmHg, while prehypertension is recommended for SBP 130–139 mmHg and/or DBP 80–89 mmHg according to the "Chinese Hypertension Clinical Practice Guidelines" [1]. Hypertension, which is a leading cause of death globally, is associated with increasing cardiovascular (CV) risk, and by 2025, the global incidence of hypertension is expected to increase by 60%, affecting 1.56 billion people [2]. It has been reported that the monthly average costs of treating hypertension and CV diseases (CVDs) for each patient are approximately 22 US$ and 300–1000 US$, respectively [3]. Hyperlipidaemia is a major systemic disorder and an important variable risk factor for coronary heart disease, and its prevalence is closely related to an increased risk of CVD [4], which is a leading contributor to mortality worldwide. The increase in the risk of developing hypertension and hyperlipidaemia is associated with an increased CV risk through adverse effects on lipid concentrations, insulin resistance, and other cardiometabolic processes [5].

Findings from the China Health and Nutrition Survey (CHNS) show that the age-standardized prevalence of high-normal blood pressure among Chinese adults aged 18 years and older increased from 30.1% in 1991 to 43.1% in 2015 [6]. From 2005 to 2018, deaths attributable to high systolic blood pressure in China rose from 1.98 to 2.67 million, with years of life lost (YLL) increasing from 40.14 to 48.16 million [7]. A 2020–2022 study on cardiovascular risk factors in China found that 38.1% of adults aged 18 and above had dyslipidaemia. Men (46.1%) showed a higher prevalence than women (29.6%), and urban residents (38.9%) were slightly more affected than rural individuals (37.4%). High levels of LDL-C exert a considerable influence on mortality and disability rates within the Chinese population. In 2017, elevated LDL-C significantly impacted the health of the Chinese population, leading to a mortality rate of 61.08 per 100,000 and contributing to 18.16 million lost disability-adjusted life years (DALYs) [8]. Therefore, hypertension and hyperlipidaemia have emerged as major risk factors for chronic diseases in China, posing a serious threat to public health. These conditions significantly increase the incidence and mortality rates of CVD, thereby underscoring substantial public health challenges.

Obesity is prevalent globally and across all age groups and is a popular research topic. The most widely used method for classifying obesity is the body mass index (BMI) [9]. However, the BMI is not a perfect measure of obesity; the weight term in BMI does not distinguish between muscle mass and fat mass [10] and the BMI also does not distinguish between fat or lean mass, and it does not distinguish the proportion of central or peripheral fat [11]. In addition, the BMI has been proven to be related to age, sex, and, in some cases, race [12].Therefore, waist circumference (WC) and the waist-to-height ratio (WHtR) are recommended as indicators of central obesity because they are related to fat distribution [13, 14]. The major limitation associated with using WC is that it does not consider the subject’s height and weight [15] and thus may over- or underestimate obesity in taller or shorter individuals [16]. On the other hand, evidences concluded that the measures of central obesity, especially the WHtR, over the BMI for detecting the risk of developing CVD in men and women and the WHtR has been proposed as a predictor of metabolic and cardiovascular abnormalities [16–19].

In the last decade, two new anthropometric indices combining traditional measures (height, weight and WC) have been suggested as alternatives to traditional anthropometric indices. In 2013, Thomas DM et al. suggested that the body roundness index (BRI) [20], which ranges from 1 to 16, and the BRI values were 4.64 ± 1.88 (men) and 5.16 ± 2.24 (women) as predictors of visceral adiposity tissue and body fat percentage, respectively. Thomas et al. showed that the BRI improved predictions of body fat compared to traditional indices. However, Maessen reported that the BRI was a weaker predictor than established indices such as the BMI and WC [21]. In 2012, the “a body shape index” (ABSI) [22], was proposed with the intention of predicting the risk of pathologies that cannot be readily identified by the BMI. That study showed a significant association between the ABSI and the amount of abdominal adipose tissue, and the ABSI appeared to be more strongly associated with premature death than WC and the BMI were [10]. However, the above studies did not have a sufficient sample size to explore the epidemiological characteristics of CV risk in different age or sex groups, or do not combine comprehensive anthropometric indices to elucidate their relationship with hypertension and hyperlipidaemia.

In this study, we evaluated the epidemiological characteristics of 65,231 Chinese residents aged 18–80 years based on data from the Thyroid Disorders, Iodine Status and Diabetes Epidemiology (TIDE) study, a national epidemiological cross-sectional study that was conducted from 2015 to 2017 and covered all 31 provinces of mainland China. This study enabled the comparison of the strength of the associations between these indices and hypertension and hyperlipidaemia.

## Materials and methods

### Sample collection

All the data analysed in this study were derived from the Thyroid Disease, Iodine Nutrition and Diabetes Epidemiology (TIDE) Study. Briefly, TIDE is a multistage, stratified sampling method used to select one representative city per province according to the population size and economic level from all 31 provinces within China. The samples were selected according to age, sex, and the ratio of urban/rural residents in each region based on the 2010 national census. The Ethics Committee of China Medical University approved the research plan. After receiving a detailed explanation of the protocol, all respondents signed informed consent forms.

### Inclusion and exclusion criteria

**Fig. 1:**
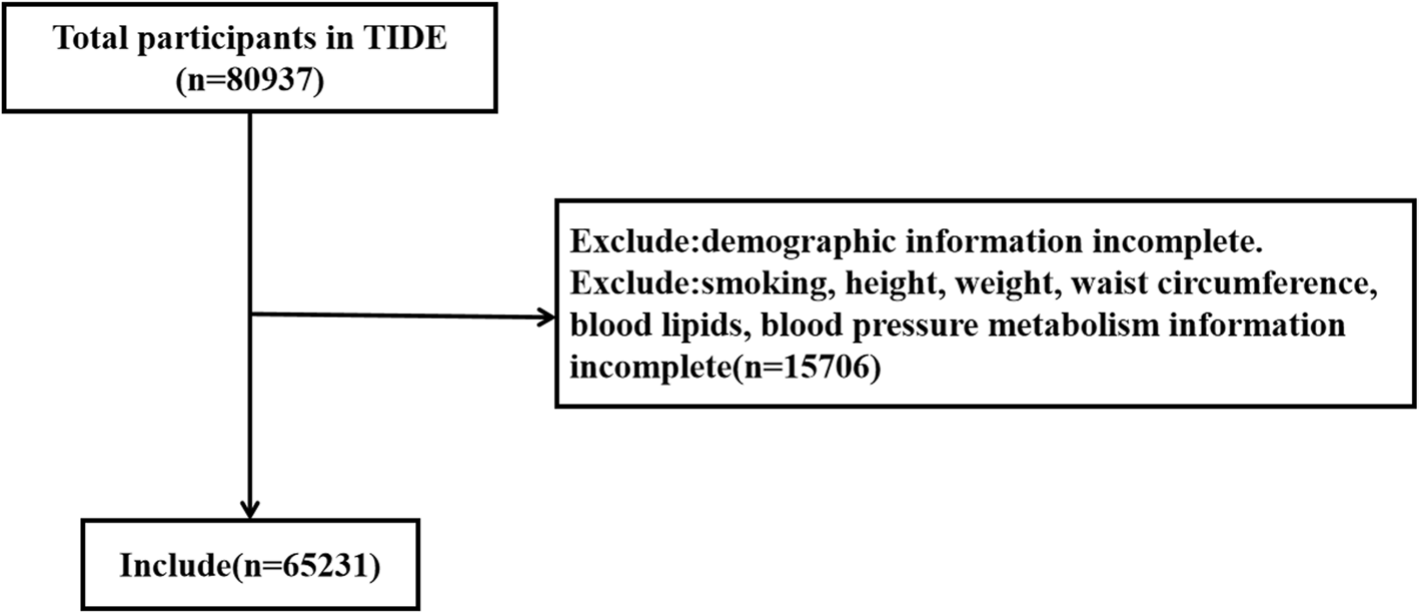
TIDE, Thyroid Disorders, Iodine Status and Diabetes, a national epidemiological cross-sectional study

The inclusion criteria were as follows: aged > 18 years; had resided locally for at least 5 years; had not taken iodine-containing drugs or contrast agents within 3 months of participation; and were nonpregnant. A total of 80,937 people participated in the project. The exclusion criteria were as follows: missing demographic data such as sex, age, family income, highest education level, ethnicity, urban and rural areas, smoking, and missing metabolic information such as height, weight, WC, blood lipid concentrations, and BP. Finally, 65,231 adults were included in the analysis. The flowchart of patient inclusion is shown in Fig. 1.

### Biochemical measurements

The collected serum and urine samples were stored at -20 °C. After investigation and specimen collection, all the samples were transported via a cold chain system to the central laboratory for unified testing. Body weight and height were measured while the study subjects were wearing light clothing and no shoes. BP (systolic blood pressure (SBP) and diastolic blood pressure (DBP)) was measured twice on the nondominant arm with a properly sized cuff using an electronic BP measuring device (Omron HEM-7430, Omron Corporation, Kyoto, Japan). All individuals were asked to empty their bladder and rest for at least 5 min before the BP measurement, and conversation was not allowed during the measurement. The mean value of two sequential measurements (separated by 10 min) was considered the final BP value. Serum triglyceride (TG), total cholesterol (TC), low-density lipoprotein cholesterol (LDL-C), and high-density lipoprotein cholesterol (HDL-C) levels were assessed using an automated biochemical analyser (MarireBS-180 analyser). Demographic information (age, sex, nationality, and location), personal and family history of thyroid diseases, and smoking status were collected using a standard questionnaire [23].

### Definitions

Weight and height were measured to the nearest 0.5 kg and 0.1 cm, respectively, with the participants wearing lightweight clothing. At the end of normal expiration, WC was measured at the umbilicus using a nonelastic tape to the nearest 0.1 cm while the participants were standing. The BMI was calculated as weight in kilograms divided by the square of the height in metres (kg/m^2^). The WHtR was calculated by dividing WC by height. The ABSI was calculated using the following formula: WC/(BMI^2/3^ × Height^1/2^) [22]. The BRI was based on height (cm) and WC (cm) and was calculated using the following formula: BRI = 364.2 − 365.5 × {1 − [(WC/2π)/ (0.5 × height)]^2^} ^0.5^ [21].

According to the Chinese guidelines on the prevention and treatment of dyslipidaemia in adults (2007) [24], hyperlipidaemia was defined as TC ≥ 5.18 mmol/L, LDL-C ≥ 3.37 mmol/L, HDL-C < 1.04 mmol/L, TG ≥ 1.70 mmol/L, and/or a history of hyperlipidaemia in the past year. According to the 2020 ISH guidelines, hypertension was defined as an SBP of 140 mmHg or greater, a DBP of 90 mmHg or greater, or the self-reported use of antihypertensive medication within the previous two weeks [25].

### Statistical analysis

Statistical analysis was completed using R software (mainly using survey packages suitable for complex sampling data analysis). The categorical variables are expressed as percentages and 95% confidence intervals (CIs) and were determined by χ2 tests or Fisher's exact tests, depending on the situation. Continuous variables are described using means and standard errors and were analysed for variance. To obtain national estimates, all calculations were based on 2010 Chinese census data, using weighting factors and the sampling scheme of the current survey, which included individuals aged 18 years and over. The weighting coefficient was the reciprocal of the adjusted probability of the respondent obtaining the data. Each case in the analysis was assigned a coefficient (individual weight) that was multiplied to represent the actual population with the same characteristics of sex, age, province, and location. Standard errors were calculated using appropriate statistical techniques and data from complex survey designs. In adjusted Model I, for the overall population, adjustments were made for sex, age, education, income, smoking status, urban or rural residence, and ethnicity. For sex-specific subgroups, the adjustments included age, education, income, smoking status, urban or rural residence, and ethnicity. In adjusted model II, we further adjusted for salt intake and diabetes. The discriminatory ability of the BMI, WC, the WHtR, the BRI and the ABSI for hypertension and hyperlipidaemia was determined by calculating the area under the curve (AUC); conducting multivariate logistic regression analysis; and calculating the corresponding 95% CIs. In adjusted models, we used restricted cubic splines (RCSs) to fit linear regression or logistic regression models to analyse the relationships between each anthropometric index and BP or blood lipid concentrations.

## Results

### General characteristics of the TIDE participants

As shown in Table 1, a total of 65,231 subjects (31,192 males and 34,039 females) were included in the study. The baseline characteristics of the study population were described based on sex and age stratification. Under different age classifications, in the 20–39-yearand 40–59-year age groups, TG (1.67, 1.93 vs. 1.08, 1.43 mmol/L; respectively), TC (4.59, 5.01 vs. 4.35, 4.94 mmol/L; respectively) and LDL-C concentrations (2.78, 3.04 vs. 2.46, 2.91 mmol/L; respectively) were greater in men than in women, and the opposite trend was observed in the 60–80-year age group (*p* < 0.0001). The SBP increased with age in both males and females. Interestingly, among males, the prevalence of hypertriglyceridaemia (40.63%), hypercholesterolaemia (39.28%), high LDL-C concentrations (29.46%) and low HDL-C concentrations (15.48%) was the highest in the 40–59-year age group, while the 60–80-year age group of women had the highest prevalence of hypertension (37.39%), hypertriglyceridaemia (31.49%), hypercholesterolaemia (51.65%), and high LDL-C concentrations (35.18%) (*p* < 0.0001).

**Table 1:** Descriptive characteristics of the study population based on age and gender

**Variable**	**Men**						**Women**						***P*** **for sex**
	**total** **(*N*** **= 31,192)**	**20–39 (*N*** **= 13,680)**	**40–59 (*N*** **= 11,490)**	**60–80 (*N*** **= 6022)**	***P*** **value**	*P* **for trend**	**Total** **(*N*** **= 34,039)**	**20–39(*N*** **= 14,571)**	**40–59(*N*** **= 12,007)**	**60–80(*N*** **= 7461)**	***P*** **value**	***P*** **for trend**	
**Age (years)**	39.00(29.00,50.00)	29.00(24.00,35.00)	47.00(43.00,52.00)	67.00(63.00,72.00)	< 0.0001	< 0.0001	39.00(29.00,49.00)	29.00(24.00,35.00)	47.00(43.00,52.00)	67.00(62.00,72.00)	< 0.0001	< 0.0001	0.06
**TG (mmol/L)**	1.74(0.02)	1.67(0.03)	1.93(0.02)	1.43(0.03)	< 0.0001	0.78	1.27(0.01)	1.08(0.01)	1.43(0.01)	1.58(0.02)	< 0.0001	< 0.0001	< 0.0001
**TC (mmol/L)**	4.78(0.01)	4.59(0.01)	5.01(0.01)	4.92(0.02)	< 0.0001	< 0.0001	4.68(0.01)	4.35(0.01)	4.94(0.01)	5.29(0.02)	< 0.0001	< 0.0001	< 0.0001
**LDL-C (mmol/L)**	2.89(0.01)	2.78(0.01)	3.04(0.01)	2.95(0.02)	< 0.0001	< 0.0001	2.70(0.01)	2.46(0.01)	2.91(0.01)	3.16(0.02)	< 0.0001	< 0.0001	< 0.0001
**HDL-C (mmol/L)**	1.38(0.00)	1.35(0.01)	1.38(0.01)	1.51(0.01)	< 0.0001	< 0.0001	1.58(0.00)	1.58(0.00)	1.57(0.00)	1.59(0.01)	0.11	< 0.0001	< 0.0001
**SBP (mmHg)**	126.28(0.13)	123.78(0.18)	127.49(0.20)	133.66(0.41)	< 0.0001	< 0.0001	118.42(0.12)	112.74(0.14)	121.72(0.19)	133.43(0.43)	< 0.0001	< 0.0001	< 0.0001
**DBP (mmHg)**	79.19(0.10)	77.69(0.14)	81.19(0.15)	79.57(0.28)	< 0.0001	< 0.0001	74.51(0.08)	72.62(0.11)	76.27(0.12)	77.16(0.27)	< 0.0001	< 0.0001	< 0.0001
**Hypertension (%)**					< 0.0001	< 0.0001					< 0.0001	< 0.0001	< 0.0001
** NO**	76.15(74.99,77.31)	82.65(81.67,83.63)	72.00(70.88,73.11)	60.26(58.15,62.37)			86.47(85.43,87.52)	94.16(93.66,94.66)	83.16(82.25,84.07)	62.21(60.04,64.37)			
** YES**	23.85(23.10,24.60)	17.35(16.37,18.33)	28.00(26.89,29.12)	39.74(37.63,41.85)			13.53(12.99,14.06)	5.84(5.34, 6.34)	16.84(15.93,17.75)	37.79(35.63,39.96)			
**Hypertriglyceridemia (%)**					< 0.0001	0.202					< 0.0001	< 0.0001	< 0.0001
** NO**	66.13(65.00,67.25)	68.51(67.32,69.70)	59.37(58.14,60.59)	76.78(75.02,78.55)			81.32(80.27,82.37)	87.97(87.25,88.69)	76.11(75.13,77.10)	68.51(66.47,70.54)			
** YES**	33.87(32.99,34.75)	31.49(30.30,32.68)	40.63(39.41,41.86)	23.22(21.45,24.98)			18.68(18.08,19.28)	12.03(11.31,12.75)	23.89(22.90,24.87)	31.49(29.46,33.53)			
**Hypercholesterolemia (%)**					< 0.0001	< 0.0001					< 0.0001	< 0.0001	< 0.0001
** NO**	68.71(67.58,69.85)	75.70(74.60,76.81)	60.72(59.49,61.95)	62.72(60.65,64.80)			73.14(72.10,74.18)	85.37(84.58,86.17)	63.89(62.77,65.00)	48.35(46.14,50.55)			
** YES**	31.29(30.43,32.14)	24.30(23.19,25.40)	39.28(38.05,40.51)	37.28(35.20,39.35)			26.86(26.16,27.57)	14.63(13.83,15.42)	36.11(35.00,37.23)	51.65(49.45,53.86)			
**High LDL-C** **(%)**					< 0.0001	< 0.0001					< 0.0001	< 0.0001	< 0.0001
** NO**	75.44(74.26,76.62)	79.42(78.44,80.41)	70.54(69.40,71.68)	73.12(71.23,75.01)			82.36(81.30,83.41)	91.08(90.47,91.69)	75.73(74.73,76.72)	64.82(62.73,66.91)			
** YES**	24.56(23.83,25.29)	20.58(19.59,21.56)	29.46(28.32,30.60)	26.88(24.99,28.77)			17.64(17.07,18.22)	8.92(8.31, 9.53)	24.27(23.28,25.27)	35.18(33.09,37.27)			
**Low HDL-C (%)**					< 0.0001	0.01					< 0.001	< 0.0001	< 0.0001
** NO**	85.73(84.62,86.85)	85.49(84.44,86.54)	84.52(83.59,85.45)	90.66(89.42,91.89)			74.83(73.82,75.84)	76.00(75.00,76.99)	73.32(72.26,74.37)	74.68(72.79,76.57)			
** YES**	14.27(13.56,14.97)	14.51(13.46,15.56)	15.48(14.55,16.41)	9.34(8.11,10.58)			25.17(24.43,25.91)	24.00(23.01,25.00)	26.68(25.63,27.74)	25.32(23.43,27.21)			
**Income (%)**					< 0.0001	< 0.0001					< 0.0001	< 0.0001	< 0.0001
** < 3000 yuan**	39.71(38.73,40.70)	31.79(30.54,33.04)	42.57(41.33,43.81)	66.06(64.11,68.00)			45.45(44.51,46.40)	37.96(36.84,39.08)	48.63(47.45,49.81)	69.32(67.41,71.23)			
** ≥ 3000 yuan**	60.29(59.23,61.34)	68.21(66.96,69.46)	57.43(56.19,58.67)	33.94(32.00,35.89)			54.55(53.64,55.45)	62.04(60.92,63.16)	51.37(50.19,52.55)	30.68(28.77,32.59)			
**Education (%)**					< 0.0001	< 0.0001					< 0.0001	< 0.0001	< 0.0001
** Highschool orbelow**	62.43(61.35,63.52)	46.18(44.89,47.47)	76.20(75.13,77.26)	91.51(90.42,92.59)			63.18(62.22,64.14)	43.39(42.27,44.51)	80.47(79.57,81.37)	95.13(94.32,95.94)			
** University or above**	37.57(36.62,38.52)	53.82(52.53,55.11)	23.80(22.74,24.87)	8.49(7.41, 9.58)			36.82(35.95,37.69)	56.61(55.49,57.73)	19.53(18.63,20.43)	4.87(4.06, 5.68)			
**Location (%)**					< 0.0001	< 0.0001					< 0.0001	< 0.0001	0.39
** Urban areas**	51.25(50.28,52.23)	55.91(54.61,57.21)	48.92(47.67,50.16)	37.82(35.83,39.81)			51.88(50.96,52.80)	55.92(54.80,57.05)	49.70(48.53,50.87)	40.62(38.52,42.72)			
** Rural areas**	48.75(47.67,49.83)	44.09(42.79,45.39)	51.08(49.84,52.33)	62.18(60.19,64.17)			48.12(47.19,49.05)	44.08(42.95,45.20)	50.30(49.13,51.47)	59.38(57.28,61.48)			
**Ethnic (%)**					0.65	0.004					0.01	0.028	0.1
** Ethnicity Han**	95.17(93.97,96.38)	95.06(94.66,95.46)	95.33(94.92,95.73)	95.18(94.44,95.92)			95.46(94.40,96.52)	95.44(95.10,95.78)	95.80(95.46,96.13)	94.40(93.51,95.29)			
** Others**	4.83(4.56, 5.09)	4.94(4.54,5.34)	4.67(4.27,5.08)	4.82(4.08,5.56)			4.54(4.31, 4.77)	4.56(4.22,4.90)	4.20(3.87,4.54)	5.60(4.71,6.49)			
**Smoke (%)**					< 0.0001	< 0.0001					< 0.0001	< 0.0001	< 0.0001
** NO**	56.03(54.94,57.13)	61.68(60.44,62.93)	49.26(48.02,50.51)	52.18(50.05,54.32)			98.56(97.52,99.60)	98.88(98.68,99.08)	98.51(98.26,98.76)	97.25(96.63,97.88)			
** YES**	43.97(43.02,44.91)	38.32(37.07,39.56)	50.74(49.49,51.98)	47.82(45.68,49.95)			1.44(1.28, 1.59)	1.12(0.92,1.32)	1.49(1.24,1.74)	2.75(2.12,3.37)			
**Salt intake (%)**					< 0.0001	< 0.0001					< 0.0001	< 0.0001	< 0.0001
** Severe**^**a**^	19.58(19.04,20.13)	16.90(16.10,17.70)	22.92(22.04,23.80)	20.96(19.69,22.23)			16.07(15.56,16.59)	13.69(12.95,14.43)	18.98(18.14,19.82)	17.17(15.97,18.36)			
** Moderate**^**b**^	60.36(59.38,61.34)	63.31(62.11,64.51)	59.17(58.13,60.22)	56.77(55.20,58.33)			58.70(57.74,59.67)	62.40(61.28,63.52)	57.35(56.29,58.40)	52.74(51.14,54.34)			
** Mild**^**c**^	19.34(18.64,20.04)	19.79(18.69,20.89)	17.91(17.08,18.73)	22.27(20.95,23.59)			24.68(24.01,25.36)	23.91(22.91,24.91)	23.68(22.76,24.59)	30.09(28.62,31.57)			
**Diabetes (%)**					< 0.0001	< 0.0001					< 0.0001	< 0.0001	< 0.0001
** NO**	86.01(84.91,87.11)	95.36(94.96,95.77)	79.99(79.12,80.86)	71.14(69.71,72.56)			88.01(86.96,89.07)	96.19(95.77,96.60)	86.42(85.67,87.17)	67.44(65.94,68.93)			
** YES**	13.99(13.53,14.45)	4.64(4.23, 5.04)	20.01(19.14,20.88)	28.86(27.44,30.29)			11.99(11.54,12.43)	3.81(3.40, 4.23)	13.58(12.83,14.33)	32.56(31.07,34.06)			

Data are presented as mean ± standard error (SE) or percentage. ¥30,000 = £3365; €3849; $4115

*TG* triglyceride, *TC* total cholesterol, *LDL-C* low density lipoprotein cholesterol, *HDL-C* high density lipoprotein cholesterol, *SBP* systolic blood pressure, *DBP* diastolic blood pressure

^a^Severe: salt intake greater than 10 g/person/day; ^b^Moderate: salt intake between 5–10 g/person/day; ^c^Mild: salt intake less than 5 g/person/day

### Descriptive anthropometric adiposity indices based on age and sex

Table 2 describes the anthropometric adiposity indices, such as the BMI, WC, the WHtR, the BRI, and the ABSI, according to age and sex. For both males and females, there were significant differences in the five obesity measures, the BMI, WC, the WHtR, the BRI, and the ABSI, across different age groups (*P* < 0.0001). In males, the BMI, WC, the WHtR, the and BRI were greater in the 40–59-year age group than in the other age groups, while the ABSI was greater in the 60–80-year age group than in the other age groups. Among women, the BMI, WC, the WHTR, the BRI, and the ABSI were greater in the 60–80-year age group than in the other age groups. In the 20–39-year-old age group and 40–59-year-old age group, the BMI, WC, the WHtR, the BRI, and the ABSI of men were greater than those of women. In the 60–80-year age group, the BMI, the WHtR, the BRI, and the ABSI of women were greater than those of men, except for WC.

**Table 2:** Descriptive anthropometric indices (BMI, WC, WHtR, BRI, and ABSI) of the study population based on age and gender

**Anthropometric indices**	**Men**						**Women**						***P*** **for sex**
	**total**	**20–39**	**40–59**	**60–80**	***P*** **value**	*P* **for trend**	**total**	**20–39**	**40–59**	**60–80**	***P* value**	***P*** **for trend**	
**Body mass index** **(Kg/m**^**2**^**)**	24.29(0.03)	23.99(0.05)	24.88(0.04)	23.77(0.07)	< 0.0001	< 0.0001	23.06(0.03)	22.14(0.04)	24.01(0.04)	24.04(0.08)	< 0.0001	< 0.0001	< 0.0001
**Waist circumstance** **(cm)**	85.75(0.09)	84.37(0.14)	87.70(0.12)	85.73(0.22)	< 0.0001	< 0.0001	78.50(0.08)	75.39(0.11)	81.08(0.11)	83.98(0.22)	< 0.0001	< 0.0001	< 0.0001
**Waist-to-height ratio**	0.50(0.0006)	0.49(0.0008)	0.52(0.0007)	0.52(0.0012)	< 0.0001	< 0.0001	0.50(0.0005)	0.47(0.0007)	0.51(0.0007)	0.55(0.0014)	< 0.0001	< 0.0001	< 0.0001
**Body roundness index**	3.55(0.01)	3.30(0.02)	3.83(0.01)	3.81(0.02)	< 0.0001	< 0.0001	3.37(0.01)	2.92(0.01)	3.69(0.01)	4.35(0.03)	< 0.0001	< 0.0001	< 0.0001
**A body shape index**	0.078(0.0000)	0.0777(0.0001)	0.0795(0.0001)	0.0810(0.0001)	< 0.0001	< 0.0001	0.0773(0.0001)	0.0759(0.0001)	0.0778(0.0001)	0.0817(0.0002)	< 0.0001	< 0.0001	< 0.0001

*BMI* body mass index, *WC* waist circumference, *WHtR* Waist-to-height ratio, *BRI* Body roundness index, *ABSI* A body shape index

### Associations between anthropometric indices and SBP and DBP

**Fig. 2:**
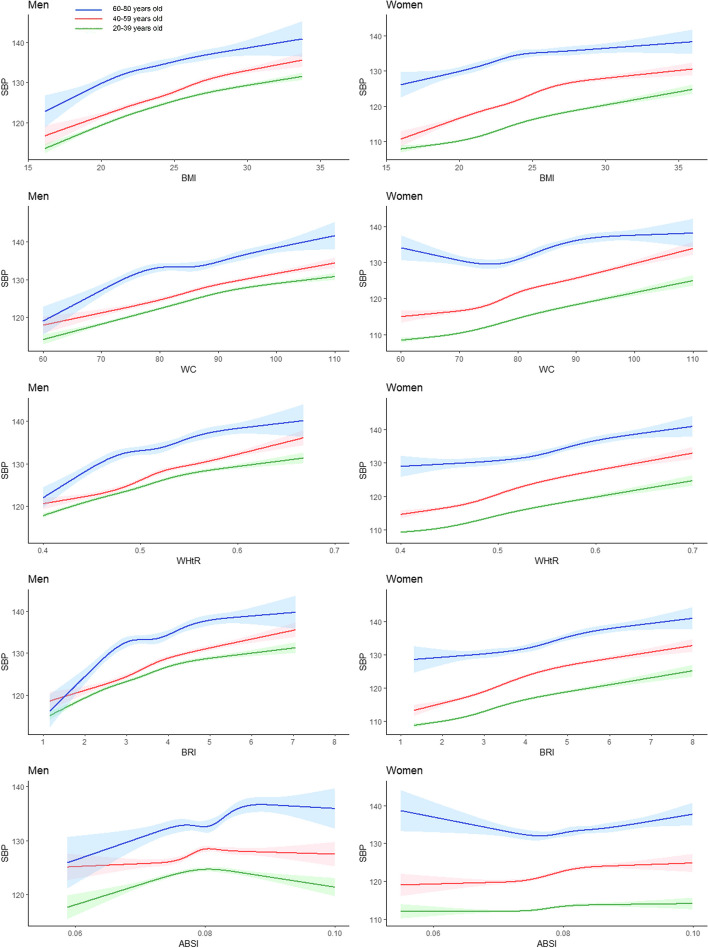
Restricted Cubic Splines of Systolic Blood Pressure Change Trend under Different Age Stratifications in Adjusted Models. The solid green line represents 20–39 years old, red represents 40–59 years old, and blue represents 60–80 years old. The solid lines in the three colours represent the merged model curves. The regions of the three colours represent 95% CIs of the combined curve for that colour

**Fig. 3:**
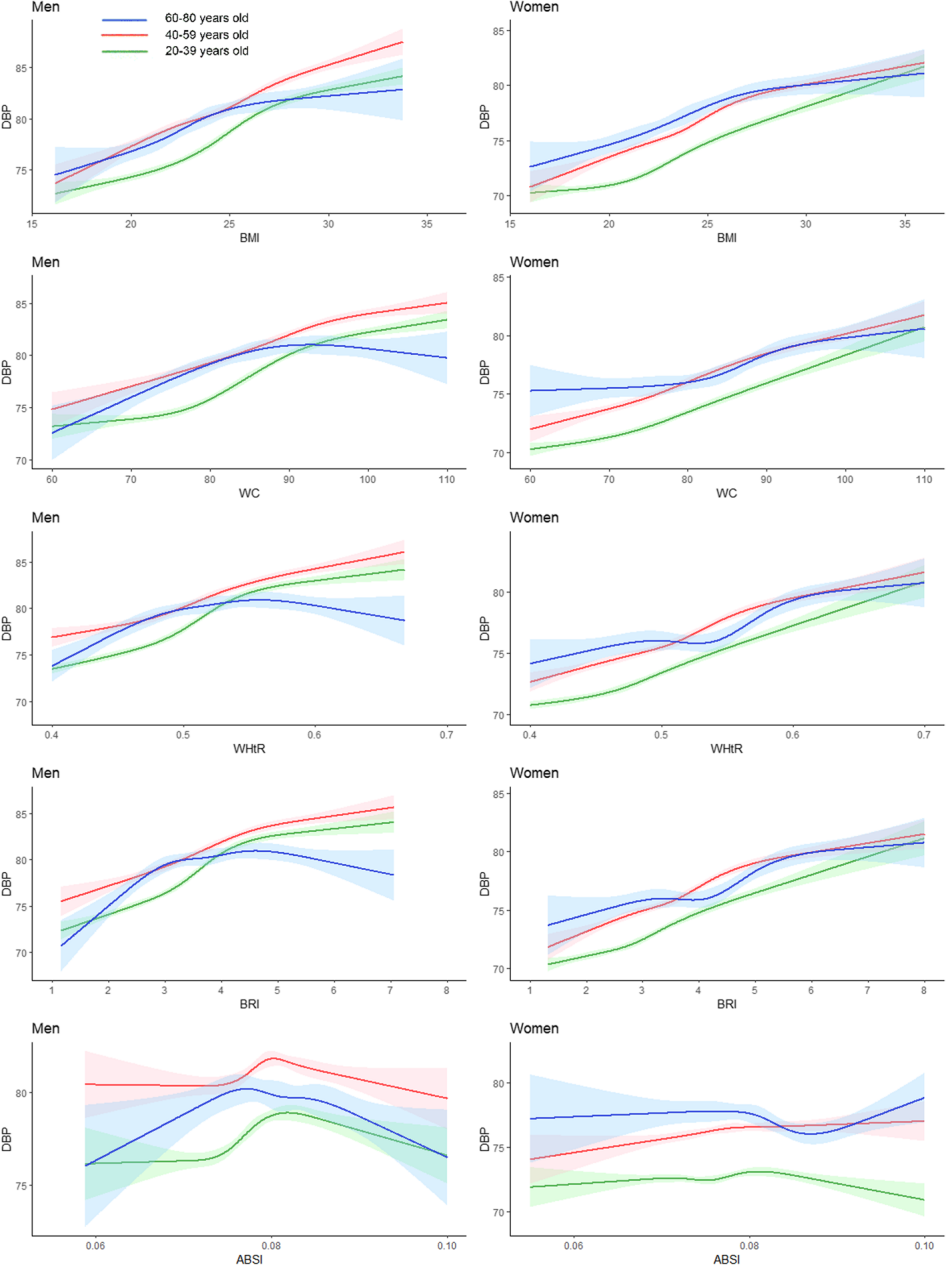
Restricted cubic splines of diastolic blood pressure change trend under different age stratifications in adjusted models. The solid green line represents 20–39 years old, red represents 40–59 years old, and blue represents 60–80 years old. The solid lines in the three colours represent the merged model curves. The regions of the three colours represent 95% CIs of the combined curve for that colour

As shown in Fig. 2, we observed an almost linear relationship between SBP and the BMI, WC, the WHtR, and the BRI in the 20–39-year and 40–59-year age groups. The SBP of the population aged 60–80-year group was highest compared to other age groups regardless of the gender. Notably, we found that the 60–80-year group showed a cubic relationship with almost all indices. For women in the 60–80-year age group who had a decreasing trend in SBP before their WC was less than 80 cm, SBP increased rapidly after their WC was greater than 80 cm, showing a U-shaped relationship. As shown in Fig. 3, among the total population, there was a positive correlation between DBP and the BMI, WC, the WHtR, and the BRI in people aged 20–39 years and 40–59 years. However, a noteworthy phenomenon was also observed in the 60–80 age group. DBP tends to stabilize in people aged 60–80 years when their BMI is over 24 kg/m^2^. Interestingly, there was an inverted U-shaped relationship between WC, the WHtR, the BRI, and DBP in the male population aged 60–80 years.

### Associations between anthropometric indices and TG, TC, LDL-C and HDL-C concentrations

**Fig. 4:**
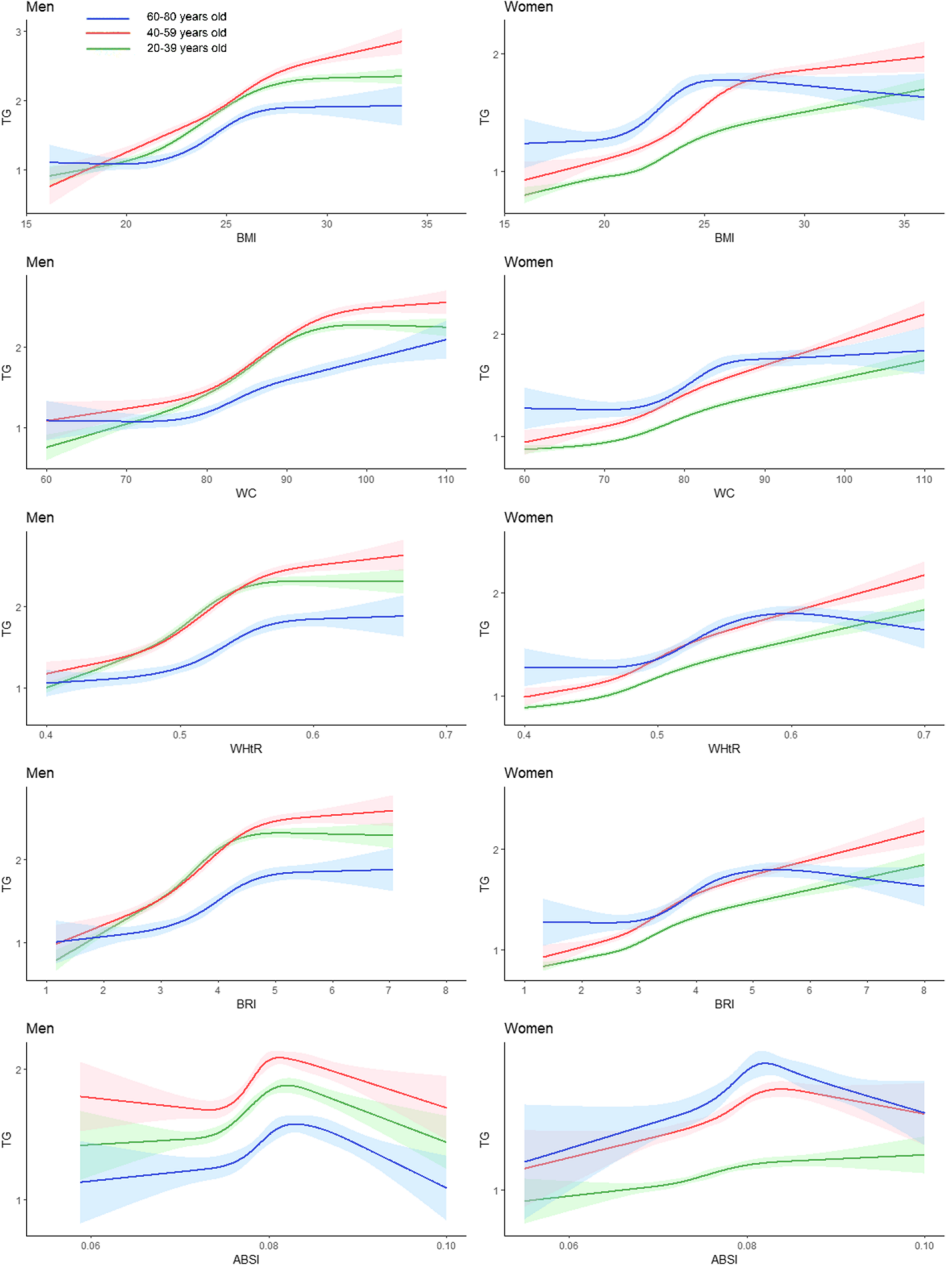
Restricted cubic splines of serum triglyceride change trend under different age stratifications in adjusted models. The solid green line represents 20–39 years old, red represents 40–59 years old, and blue represents 60–80 years old. The solid lines in the three colours represent the merged model curves. The regions of the three colours represent 95% CIs of the combined curve for that colour

Figure 4 shows that TG in the male population in all age groups increased with an increasing BMI, WC, WHtR, and BRI, and compared to that in the 60–80-year age group, the 20–39-year and 40–59-year age groups had greater TG concentrations. Among the female participants, the BMI ranged from 24 to 25, WC ranged from 80 to 85, the WHtR ranged from 0.5 to 0.6, and the BRI ranged from 3 to 4, and TG increased rapidly and then stabilized or slightly decreased in women aged 60–80 years.

**Fig. 5:**
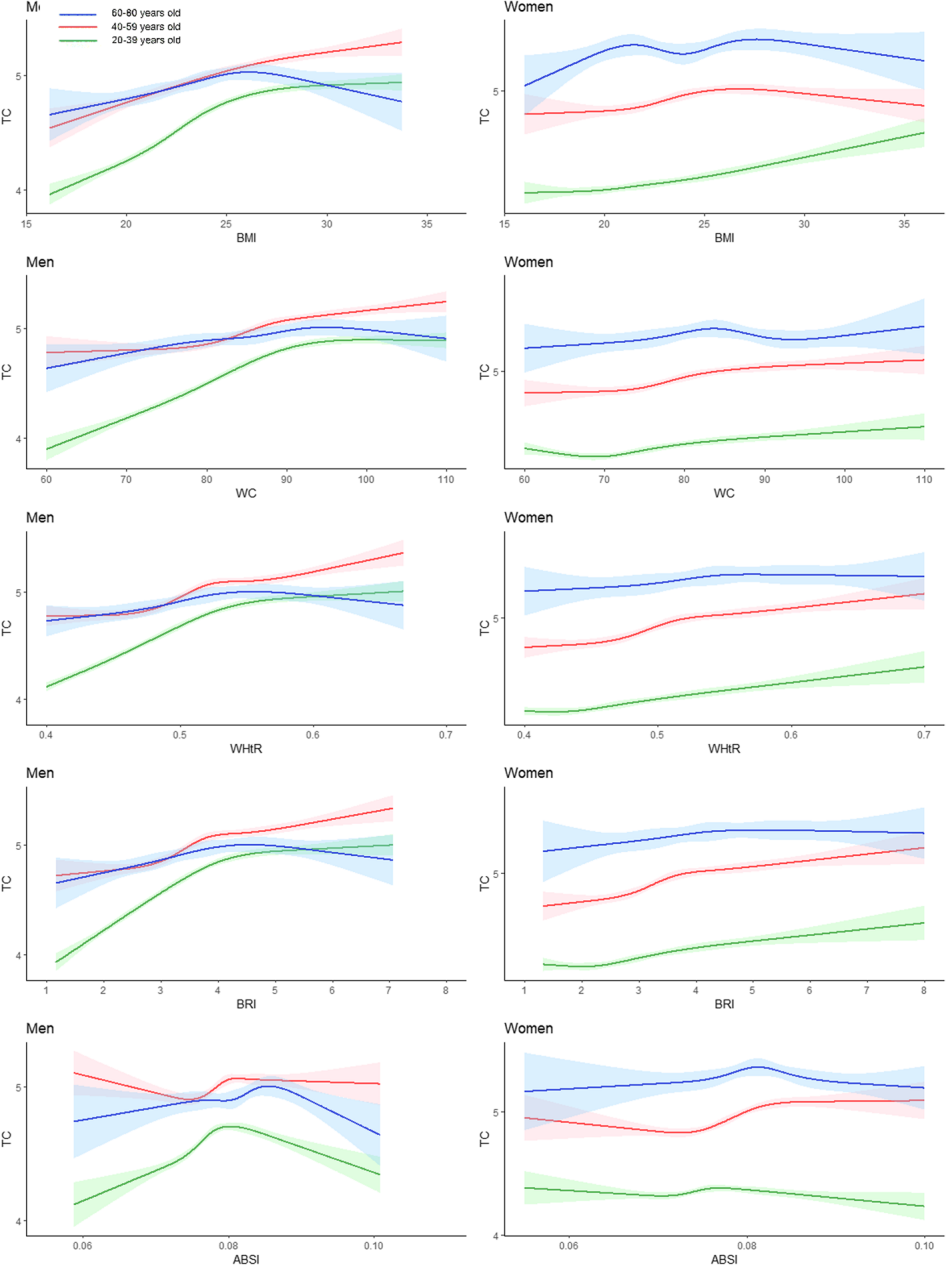
Restricted cubic splines of total serum cholesterol change trend under different age stratifications in adjusted models. The solid green line represents 20–39 years old, red represents 40–59 years old, and blue represents 60–80 years old. The solid lines in the three colours represent the merged model curves. The regions of the three colours represent 95% CIs of the combined curve for that colour

Figure 5 shows that the TC of males in the 20–39-year age group increased rapidly with increasing BMI, WC, WHtR, and BRI and then tended to stabilize. For males aged 60–80 years, TC reached its peak at a BMI of 25 years, followed by a gradual decrease in TC, forming an inverted U-shaped pattern. The changes in the TC of women in different age groups with increasing anthropometric indices were not significant, and the TC of women aged 60–80 years was greater than that of women in other age groups. Interestingly, we found that the TC and BMI of women aged 60–80 years exhibited an M-shaped pattern.

**Fig. 6:**
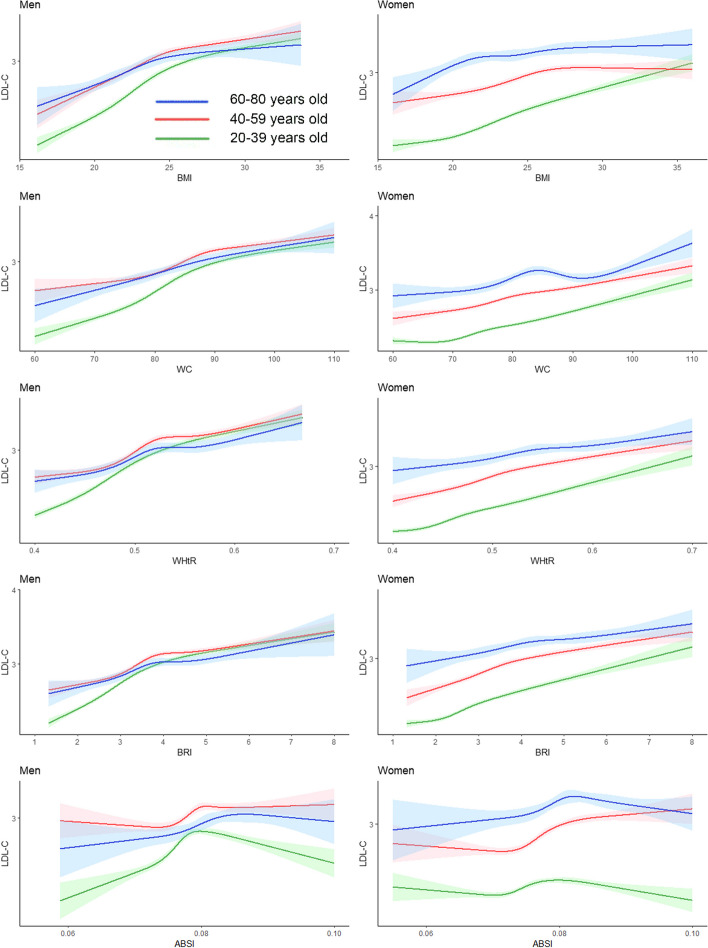
Restricted cubic splines of low-density lipoprotein cholesterol change trend under different age stratifications in adjusted models. The solid green line represents 20–39 years old, red represents 40–59 years old, and blue represents 60–80 years old. The solid lines in the three colours represent the merged model curves. The regions of the three colours represent 95% CIs of the combined curve for that colour

Figure 6 shows that there was a positive correlation between LDL-C and BMI, WC, the WHtR, and the BRI in general for both males and females. However, the trend of LDL-C changes in different age groups of men is similar, while LDL-C reaches its highest point in women aged 60–80 years at a WC of 85, followed by a downwards trend at a WC of 85–90, and finally a gradual increase again. In total, men in the 40- to 59-year-old age group had higher LDL levels than men in the other age groups, while women in the 60- to 80-year-old age group had higher LDL levels.

**Fig. 7:**
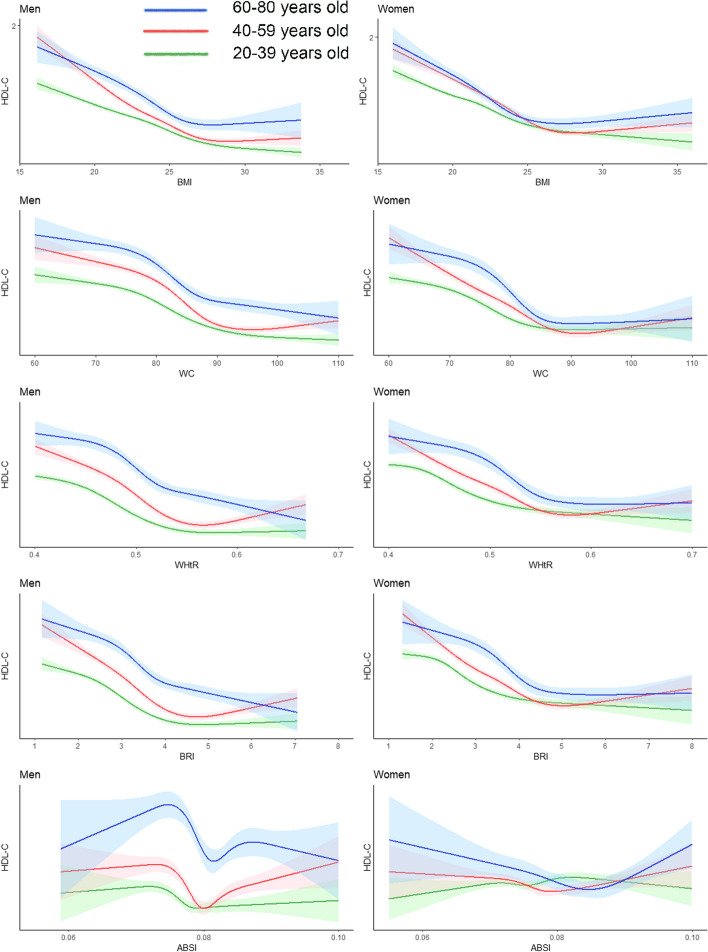
Restricted cubic splines of high-density lipoprotein cholesterol change trend under different age stratifications in adjusted models. The solid green line represents 20–39 years old, red represents 40–59 years old, and blue represents 60–80 years old. The solid lines in the three colours represent the merged model curves. The regions of the three colours represent 95% CIs of the combined curve for that colour

As shown in Fig. 7, we found a negative correlation between HDL-C and the BMI, WC, the WHtR, and the BRI, but there was no significant correlation between HDL-C concentrations and the ABSI.When BMI ranges from 22 to 25, WC ranges from 75 to 85, WHtR ranges from 0.45 to 0.5, and BRI ranges from 3 to 4, HDL-C decreases most rapidly and then gradually stabilizes.

### AUCs of anthropometric indices for the discriminatory ability of hypertension and hyperlipidaemia

Table 3 shows the AUC values [95% CIs] of the anthropometric indices used to screen for hypertension and hyperlipidaemia. In model adjusted for sex, age, education, income, smoking status, urban or rural residence, and ethnicity, when distinguishing hypertension, the AUCs of the WHtR and BRI (0.665, *p* < 0.001; 0.665, *p* < 0.001) were highest while WC (0.663, *p* < 0.001) performed only marginally lower in the overall population. In distinguishing hypertriglyceridaemia, the AUCs for the BMI (0.711, *P* < 0.001) and WC (0.715, *P* < 0.001) were greater than those of several other indicators in the general population. In distinguishing hypercholesterolaemia and high LDL-C status, both the WHtR (0.629, *P* < 0.001; 0.659, *P* < 0.001) and BRI (0.629, *P* < 0.001; 0.659, *P* < 0.001) had the highest AUCs, while for low HDL-C status, the BMI (0.631, *P* < 0.001) had the highest AUC. Furthermore, we additionally adjusted for salt intake and diabetes in model II (Table S1), and the results were in alignment with the findings from Model I. The relationships between anthropometric indices and the risk of hypertension and hyperlipidaemia were visualized using RCS. In individuals aged 20–39, we observed a more pronounced shift in odds ratios (ORs) for hypertension, hypertriglyceridemia, hypercholesterolemia, high LDL-C status, and low HDL-C status once BMI exceeded a critical threshold, compared to other age groups (Figure S1-S5).

**Table 3:** Area under curve (AUC) of each anthropometric indices for hypertension and hyperlipidaemia in male and female genders in adjusted model I

Anthropometric index	BMI		WC		WHtR		BRI		ABSI	
	**AUC**	**CI95%**	**AUC**	**CI95%**	**AUC**	**CI95%**	**AUC**	**CI95%**	**AUC**	**CI95%**
**Hypertension**										
Total	0.652	0.646–0.658	0.663	0.658–0.669	0.665^c^	0.660–0.671	0.665^c^	0.660–0.671	0.577	0.571–0.583
Male	0.625^a^	0.617–0.632	0.620^a^	0.612–0.628	0.637^c^	0.629–0.645	0.637^c^	0.629–0.645	0.546	0.537–0.554
Female	0.659	0.650–0.668	0.668	0.659–0.677	0.691^c^	0.682–0.700	0.691^c^	0.682–0.670	0.589	0.579–0.598
**Hypertriglyceridaemia**										
Total	0.711^a^	0.706–0.716	0.715^a^	0.710–0.720	0.701^c^	0.696–0.705	0.701^c^	0.696–0.705	0.578	0.573–0.584
Male	0.707^a^	0.701–0.714	0.703^a^	0.696–0.709	0.700^c^	0.694–0.707	0.700^c^	0.694–0.707	0.559	0.552–0.566
Female	0.692	0.684–0.699	0.685	0.677–0.692	0.695^c^	0.688–0.702	0.695^c^	0.688–0.702	0.570	0.562–0.578
**Hypercholesterolaemia**										
Total	0.598	0.593–0.603	0.607	0.602–0.613	0.629^c^	0.624–0.634	0.629^c^	0.624–0.634	0.575	0.570–0.580
Male	0.602^a^	0.595–0.610	0.608^a^	0.601–0.616	0.626^c^	0.619–0.634	0.626^c^	0.619–0.634	0.563	0.555–0.571
Female	0.592	0.585–0.599	0.610	0.603–0.617	0.630^c^	0.623–0.637	0.630^c^	0.623–0.637	0.582	0.575–0.590
**High LDL-C**										
Total	0.637^a^	0.632–0.643	0.652^ab^	0.646–0.657	0.659^bc^	0.653–0.664	0.659^bc^	0.653–0.664	0.586	0.581–0.592
Male	0.630^a^	0.622–0.637	0.635^a^	0.627–0.642	0.647^c^	0.639–0.654	0.647^c^	0.639–0.654	0.563	0.555–0.571
Female	0.632	0.624–0.640	0.654	0.646–0.662	0.665^c^	0.658–0.673	0.665^c^	0.658–0.673	0.596	0.588–0.604
**Low HDL-C**										
Total	0.631	0.625–0.637	0.588	0.582–0.594	0.613^c^	0.607–0.618	0.613^c^	0.607–0.618	0.497	0.491–0.503
Male	0.666	0.657–0.675	0.651	0.642–0.660	0.640^c^	0.631–0.649	0.640^c^	0.631–0.649	0.518	0.508–0.527
Female	0.644	0.637–0.651	0.619	0.612–0.627	0.613^c^	0.606–0.621	0.613^c^	0.606–0.621	0.508	0.501–0.516

Groups that share the same superscript letter do not exhibit any statistical difference between them. Conversely, a superscript with no letter indicates that the group is statistically different from all other groups

Model I: For the overall population, adjustments were made for sex, age, education, income, smoking status, urban or rural residence, and ethnicity. For sex-specific subgroups, the adjustments included age, education, income, smoking status, urban or rural residence, and ethnicity

## Discussion

Considering that both hypertension and hyperlipidaemia are very common and interrelated with each other and public health problems among adults in China, determining the relationships among BP, blood lipid concentrations, and anthropometric measurements, is critical.

In this study, the AUC was used to assess the feasibility of using anthropometric indices to screen for hypertension and hyperlipidaemia. With age stratification, RCS fitting for generalized linear regression was used to investigate the relationships between BP or blood lipid concentrations and the BMI, WC, the WHtR, the BRI and the ABSI, which are feasible and strong indicators. The RCS with age stratification showed that SBP and DBP levels were significantly correlated with the BMI, WC, the WHtR, and the BRI, and these results were consistent with those of previous studies [26–28]. Moreover, when investigating the correlation between BP and anthropometric indices, we identified a noteworthy phenomenon that the 60–80-year group showed a cubic relationship with almost all indices. There was a U-shaped relationship between SBP and WC in the female population aged 60–80 years. Despite the decline in estrogen levels after menopause, women with smaller WC (< 80 cm) and a higher proportion of subcutaneous fat may still derive small amounts of estrogen from subcutaneous adipose tissue, which provides some protection against elevated BP. However, when the WC exceeds 80 cm, the detrimental effects of visceral fat become more pronounced, compromising BP regulation [29, 30]. Besides, obesity in older men significantly elevates cardiac workload and often leads to diastolic dysfunction [31]. This condition impairs the heart's filling capacity during diastole, which may cause an inverted U-shaped relationship between DBP and WC, the WHtR, the BRI in 60–80-year male group.

In the model I adjusted for sex, age, education, income, smoking status, urban or rural residence, and ethnicity, the WHtR and BRI (0.665, *p* < 0.001 and 0.665, *p* < 0.001, respectively) were found to be better in screening hypertension regardless of sex among Chinese adults, and the estimated AUCs for the WHtR and BRI for hypertension were greater than those for WC, the BMI and the ABSI. This was consistent with previous studies. A systematic review including 78 different studies [32] and a meta-analysis including 10 studies reported that the WHtR (0.684 for male; 0.732 for female) is a better significant predictor of SBP than BMI (0.617 for male; 0.693 for female) and WC (0.669 for male; 0.715 for female) are [19]. In addition, some studies carried out in Korea [33], Jordan [34], and China [27] showed that WC, WHR, and WHtR were stronger predictors of hypertension risk than BMI. Currently, a WHtR < 0.5 has been established as a reference value for preventing hypertension and decreasing BP in hypertensive patients [32].

Compared to the above studies, our study introduces two new indicators: the BRI and the ABSI. The BRI calculation primarily involves WC and height and is predominantly used to evaluate obesity distribution in humans [20]. The ABSI calculation incorporates several BRI variables as well as BMI, another measure used to assess human obesity distribution [22]. However, there is a distinction between the two: the BRI is more commonly used to evaluate an individual’s overall physical fitness, while the ABSI is more commonly used to reflect the health implications of abdominal obesity. Our study suggested that the BRI had a greater discriminatory ability than the ABSI for hypertension, which was similar to the results of a previous study. A study from China reported a significant nonlinear positive dose‒response relationship between all anthropometric measures and hypertension across sexes, except for the ABSI (p nonlinearity < 0.05), which includes the BRI [35]. This study, however, was limited to a target population from only one province of China aged over 65 years. A study by Anto et al. revealed that after adjusting for all variables, the odds ratio of ABSI on the risk of metabolic syndrome was not statistically significant (*p* > 0.05), while that of BRI remained significant (*p* < 0.05) [36]. Similarly, when identifying metabolic disorders in both adult and paediatric populations in China, the BRI was found to possess superior predictive power compared to the ABSI [37, 38]. All of these findings suggest that the BRI has greater predictive value than the ABSI in predicting metabolic disorders.

Notably, obese individuals often exhibit insulin resistance and lipoprotein metabolism disorders, such as heightened plasma concentrations of TG-rich lipoprotein residues, residue-like particulate cholesterol, and apolipoprotein B, all of which are more pronounced in obese individuals with hypertriglyceridaemia [39]. In adjusted model I of this study, we found that BMI (0.707, *P* < 0.001) and WC (0.703, *P* < 0.001) were better for screening hypertriglyceridaemia in men, while WHtR (0.695, *P* < 0.001) and BRI (0.695, *P* < 0.001) were better in women. For screening hypercholesterolaemia and high LDL-C status, both the WHtR (0.629, *P* < 0.001; 0.659, *P* < 0.001) and BRI (0.629, *P* < 0.001; 0.659, *P* < 0.001) had the highest AUCs, while for screening low HDL-C status, the BMI (0.631, *P* < 0.001) had the highest AUC.

A recent study [40] reported similar results. The WHtR had the strongest association with the prevalence of hypertension and hyperlipidaemia. Chen [40] further discovered that the WHtR obesity classification with a cut-off of 0.5 was very sensitive to the classification of patients with hypertension and hyperlipidaemia, far exceeding the associations between other anthropometric indices. Nevertheless, Rao [41] showed that there was a significant correlation between different BMI ranges and hyperlipidaemia. Overweight and obesity were independently associated with increased risks of hyperlipidaemia after adjustment for confounding factors. Furthermore, the dose–response analysis indicated a significant nonlinear association between BMI and the risk of developing hyperlipidaemia, with a significant increasing trend in the odds ratio per 1 kg/m^2^ increase in BMI. However, the studies above included only the BMI as an indicator and did not consider more anthropometric indicators in combination, and the study population was limited to the Northeast Region of China.

The evidence suggests that the index with the strongest association or the best predictive ability for the risk of developing hypertension and hyperlipidaemia can differ according to residence area (rural or urban), sex, cultural group, age, and ethnic group [26, 32, 33]. These results might be explained by whether participants had concomitant diseases, age, sex, race, lifestyle, or environmental factors. Additionally, these differences may arise from differences in the anthropometric indices, study designs, measurement techniques, and statistical methods used [42], resulting in variations between the findings of the present study and those of previous studies.

Due to the crucial role of hypertension, hyperlipidaemia and obesity status in the pathophysiology of the adverse effects of lipid concentrations, insulin resistance, and other cardiometabolic processes on the risk of developing CVD [43–45], our study may have public health implications because integrating anthropometric indices with BP and blood lipid concentrations for predicting metabolic disorders and CVD in humans could yield superior results. The use of combined indices, such as the BMI, WC, the WHtR and the BRI, can be included in an individual’s medical history, such as height and weight, and can be used as tools for cardiovascular health screening. Interventions for obesity are necessary for Chinese people. It is well known that healthy diets and physical activities are key to controlling the occurrence of obesity, hypertension and hyperlipidaemia. Weight reduction is recommended for hypertensive patients who are overweight or obese to control metabolic risk factors, replace trans fats with unsaturated fats, implement public awareness programs on diet and physical activity, and increase the consumption of fruits and vegetables to reduce trends in BMI and serum TC concentrations.

The survey data were nationally representative, were obtained from the TIDE project database, covered all provinces in mainland China and had a sufficient sample size and representativeness.

Clear and well-defined inclusion and exclusion criteria were established, and every effort was made to include all eligible individuals in the analysis to minimize selection bias. After weighted adjustment, this study better reflected the epidemiological characteristics of hypertension and hyperlipidaemia in Chinese residents aged ≥ 18 years. In addition, the epidemiological characteristics of each component were further analysed by age and sex. This study analysed the correlation between hypertension, hyperlipidaemia, and obesity in different age groups of the Chinese population, and our findings support that the WHtR and BRI can be used to determine hypertension and hyperlipidaemia risk in adults. Simple, cost-effective, non-invasive anthropometric measurements save time, labour, and health expenditures in the diagnosis and management of hypertension and hyperlipidaemia, and they are beneficial for national population health censuses and promotion in economically underdeveloped areas with insufficient equipment.

However, this study also has several limitations that should be addressed. First, this was a cross-sectional study, so we could not confirm the cause-and-effect relationship between hypertension, hyperlipidaemia and obesity. Because data are collected at a single point in time, cross-sectional studies cannot establish the temporal sequence of variables, which hinders the assessment of causal relationships. Furthermore, this design is unable to track changes in variables over time or identify trends and dynamic patterns in time series data, limiting its ability to evaluate long-term effects and temporal dynamics. Second, epidemiological investigations are methodologically limited by the impact of geographic, environmental, genetic, drug and other factors that may influence disease outcomes. Third, the TIDE participants were Chinese; therefore, our results may not apply to other ethnic groups, and any generalization should be considered with caution.

Moreover, this is merely a preliminary study that warrants further research. Future studies comprising many non-selected samples and long-term follow-ups are warranted to identify the role of anthropometric indices in the risk of developing prehypertension and prehyperlipidaemia. Given the partial alignment of our findings with previous studies, further investigation is warranted to determine whether these results can be generalized to populations from other cultural, ethnic, or socio-economic backgrounds. The use of combined indices, such as the BMI, WC, the WHtR and the BRI, can be included in an individual’s medical history, such as height and weight, and can be used as tools for cardiovascular health screening.

## Conclusion

In our study, several anthropometric indices were associated with hypertension and hyperlipidaemia. The WHtR and the BRI were better in screening hypertension, hypercholesterolaemia and high LDL-C status, while the BMI was better in screening hypertriglyceridaemia and low HDL-C status. Consequently, the accessibility and efficacy of anthropometric indices are superior for early warning of hypertension and hyperlipidaemia. Additionally, their noninvasive nature enhances their suitability for use in obesity-based prevention and intervention strategies for CV risk, broadening their applicability in public health initiatives.

## Supplementary Information

Additional file 1: Figure S1. Restricted cubic splines representing the ORs for hypertension under different age stratifications in adjusted models. The relationship between the anthropometric indices and ORs for hypertension. The regions of the three colours represent 95% CIs of the combined curve for that colour.

Additional file 2: Figure S2. Restricted cubic splines representing the ORs for hypertriglyceridemia under different age stratifications in adjusted models. The relationship between the anthropometric indices and ORs for hypertriglyceridemia. The regions of the three colours represent 95% CIs of the combined curve for that colour.

Additional file 3: Figure S3. Restricted cubic splines representing the ORs for Hypercholesterolemia under Different Age Stratifications in Adjusted Models. The relationship between the anthropometric indices and ORs for hypercholesterolemia. The regions of the three colours represent 95% CIs of the combined curve for that colour.

Additional file 4: Figure S4. Restricted cubic splines representing the ORs for High LDL-C status under different age stratifications in adjusted models. The relationship between the anthropometric indices and ORs for high LDL-C status. The regions of the three colours represent 95% CIs of the combined curve for that colour.

Additional file 5: Figure S5. Restricted cubic splines representing the ORs for Low HDL-C status under different age stratifications in adjusted models. The relationship between the anthropometric indices and ORs for low HDL-C status. The regions of the three colours represent 95% CIs of the combined curve for that colour.

Additional file 6: Table S1. Area under curve (AUC) of each anthropometric indices for hypertension and hyperlipidaemia in male and female genders in adjusted model II.

## Data Availability

The datasets generated for this study are available on request to the corresponding authors.

## Abbreviations

BP: Blood Pressure
CV: Cardiovascular
CVDs: Cardiovascular Diseases
BMI: Body Mass Index
WC: Waist Circumference
WHtR: Waist-to-Height Ratio
BRI: Body Roundness Index
ABSI: A Body Shape Index
TIDE: The Thyroid Disorders, Iodine Status and Diabetes Epidemiology
SBP: Systolic Blood Pressure
DBP: Diastolic Blood Pressure
TG: Triglyceride
TC: Total Cholesterol
LDL-C: Low-density Lipoprotein Cholesterol
HDL-C: High-density Lipoprotein Cholesterol
CIs: Confidence Intervals
AUC: The Area Under the Curve
RCS: Restricted Cubic Spline
