# Supplementary figures and images for: Associations between various anthropometric indices and hypertension and hyperlipidaemia: a cross-sectional study in China
Source: BMC Public Health. 2024 Nov 4;24:3045. doi: 10.1186/s12889-024-20505-w (PMC11536874; doi:10.1186/s12889-024-20505-w)

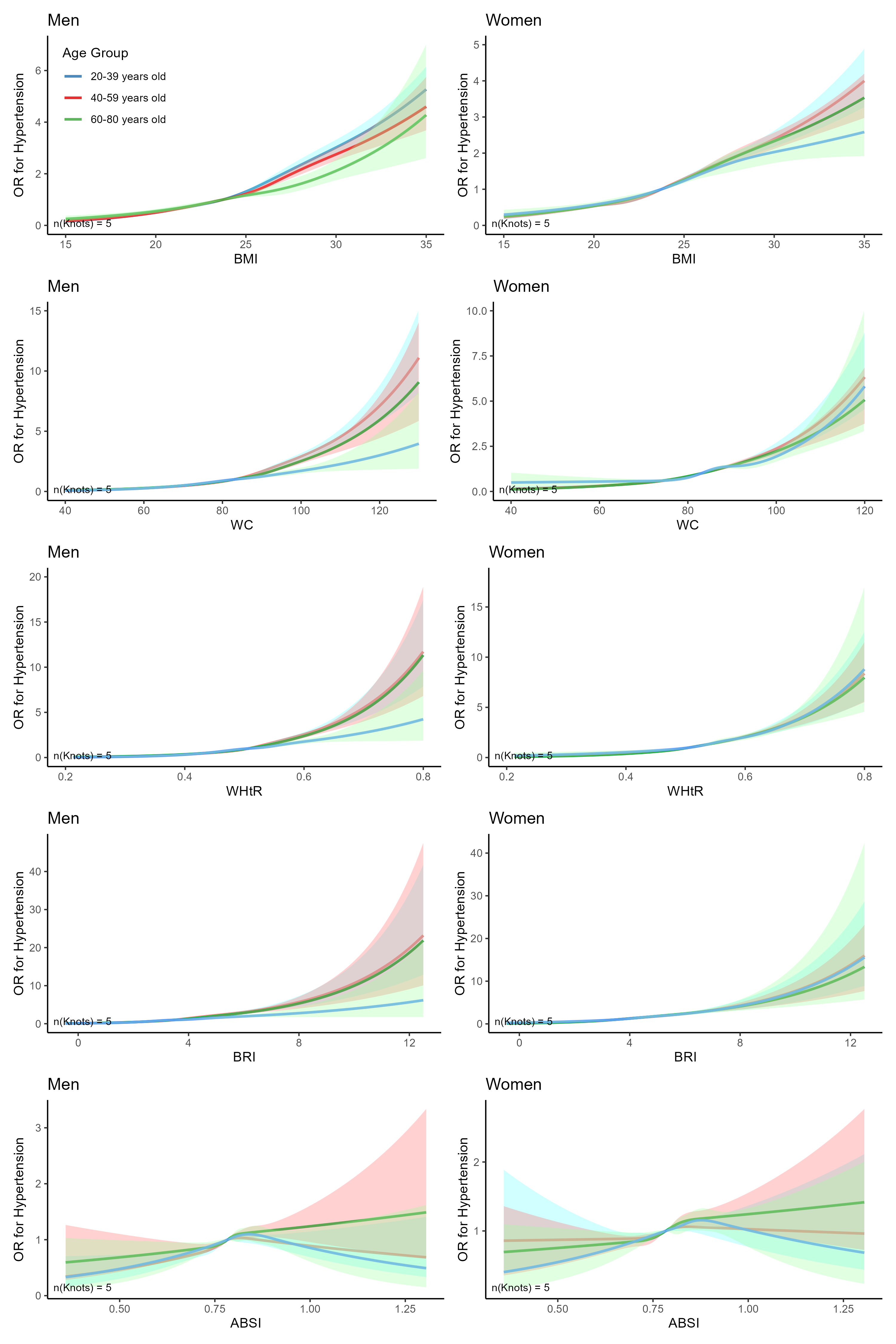

Supplement: Supplementary file 1 — Additional file 1: Figure S1. Restricted cubic splines representing the ORs for hypertension under different age stratifications in adjusted models. The relationship between the anthropometric indices and ORs for hypertension. The regions of the three colours represent 95% CIs of the combined curve for that colour. [file 12889_2024_20505_MOESM1_ESM.tiff]

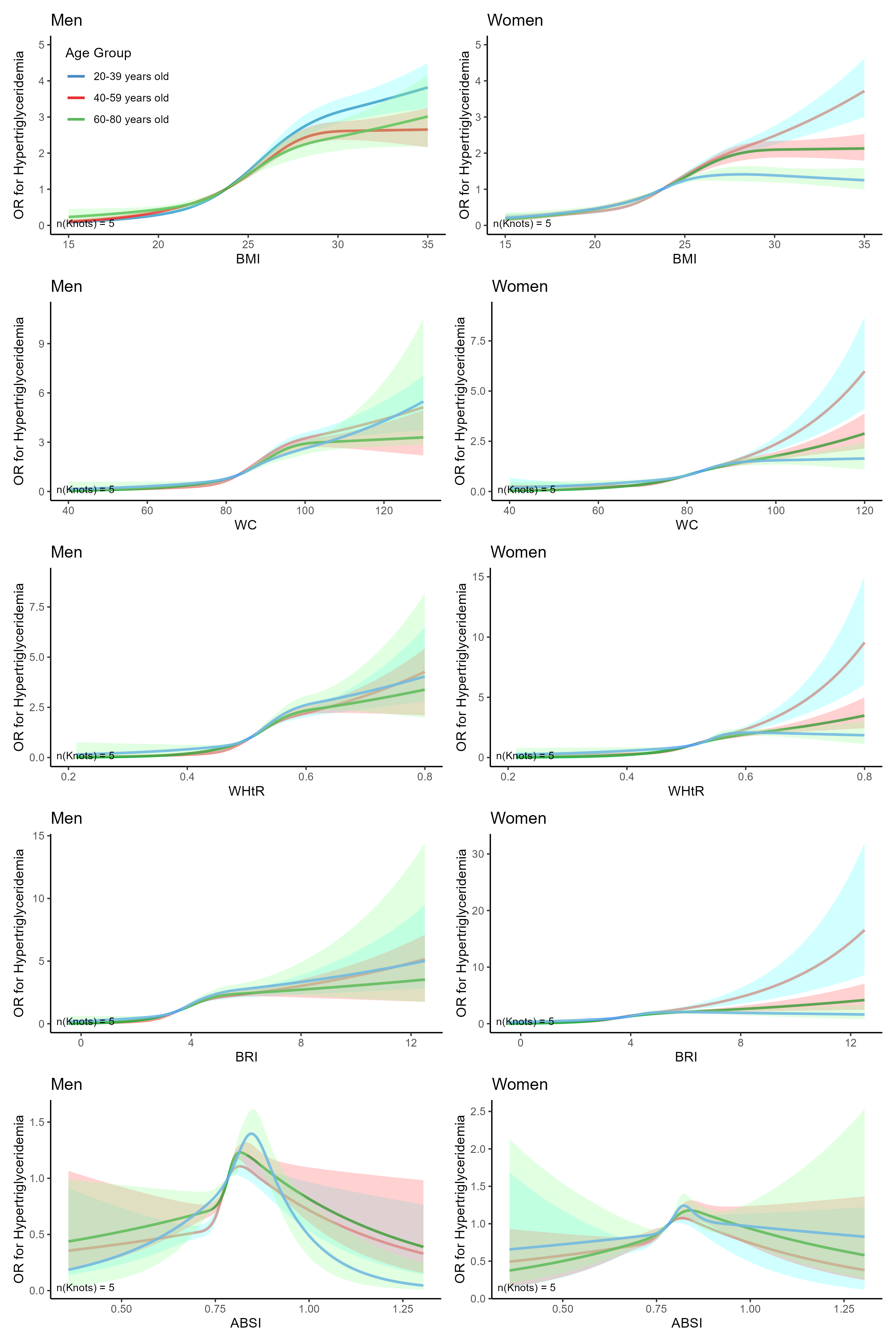

Supplement: Supplementary file 2 — Additional file 2: Figure S2. Restricted cubic splines representing the ORs for hypertriglyceridemia under different age stratifications in adjusted models. The relationship between the anthropometric indices and ORs for hypertriglyceridemia. The regions of the three colours represent 95% CIs of the combined curve for that colour. [file 12889_2024_20505_MOESM2_ESM.tiff]

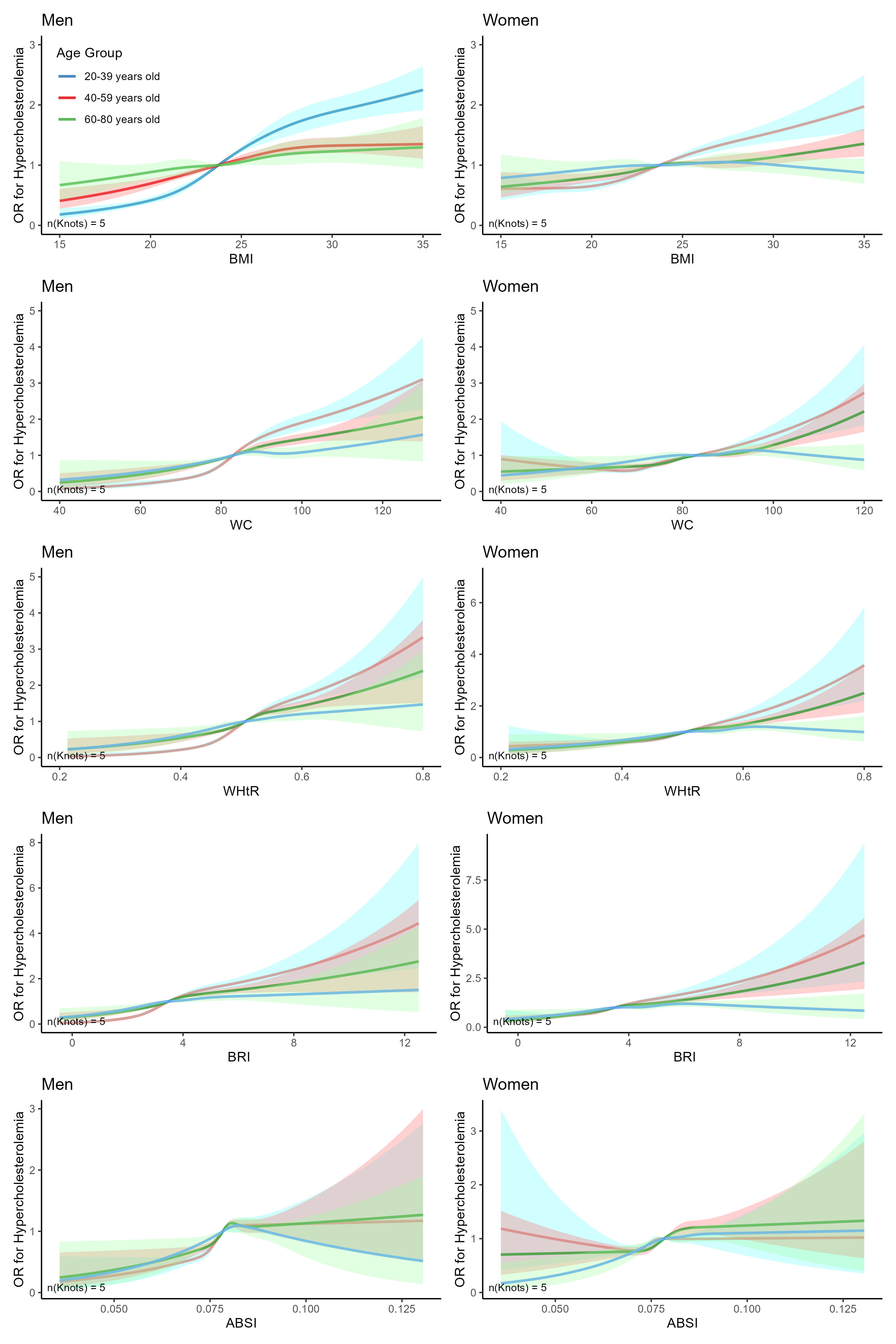

Supplement: Supplementary file 3 — Additional file 3: Figure S3. Restricted cubic splines representing the ORs for Hypercholesterolemia under Different Age Stratifications in Adjusted Models. The relationship between the anthropometric indices and ORs for hypercholesterolemia. The regions of the three colours represent 95% CIs of the combined curve for that colour. [file 12889_2024_20505_MOESM3_ESM.tiff]

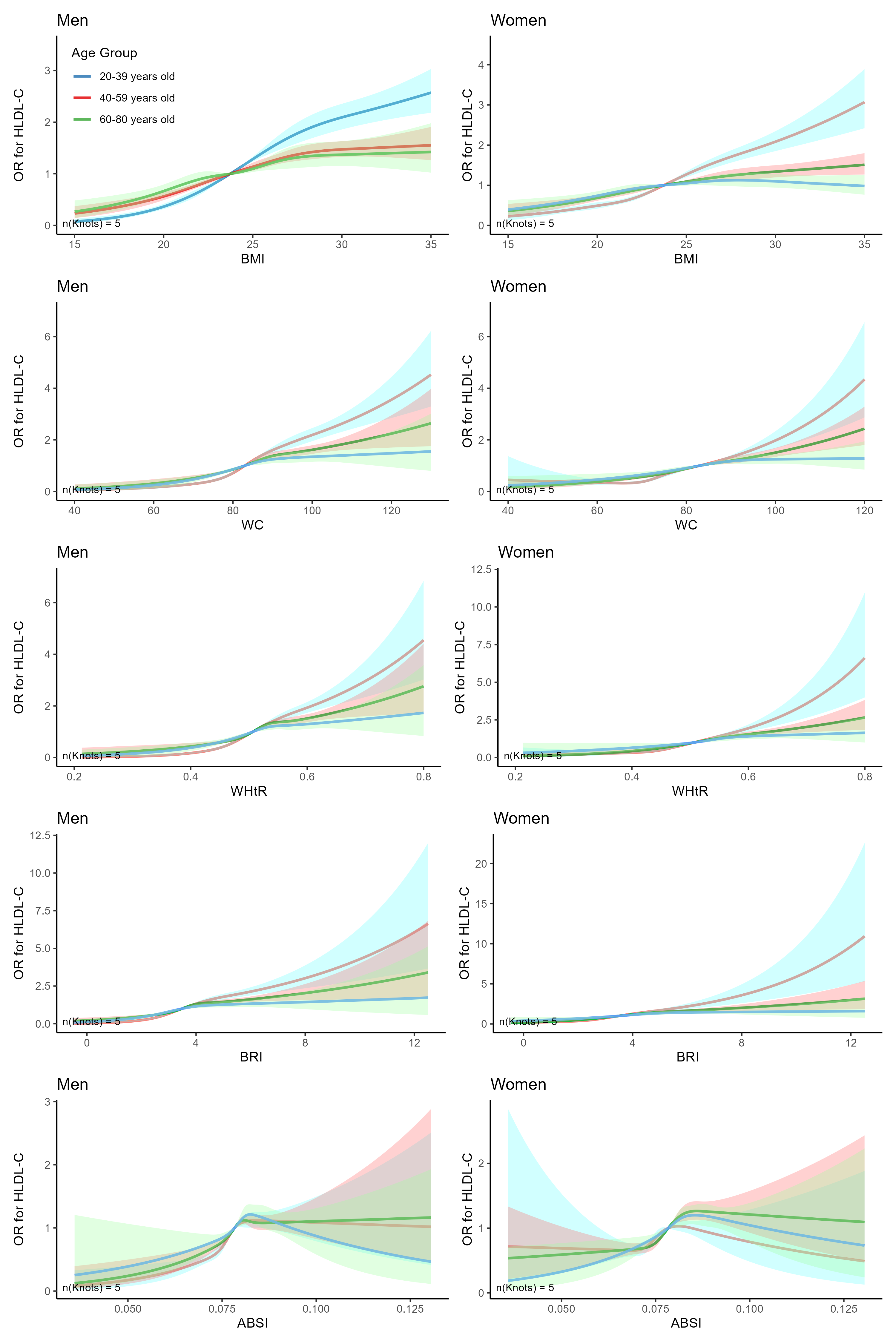

Supplement: Supplementary file 4 — Additional file 4: Figure S4. Restricted cubic splines representing the ORs for High LDL-C status under different age stratifications in adjusted models. The relationship between the anthropometric indices and ORs for high LDL-C status. The regions of the three colours represent 95% CIs of the combined curve for that colour. [file 12889_2024_20505_MOESM4_ESM.tiff]

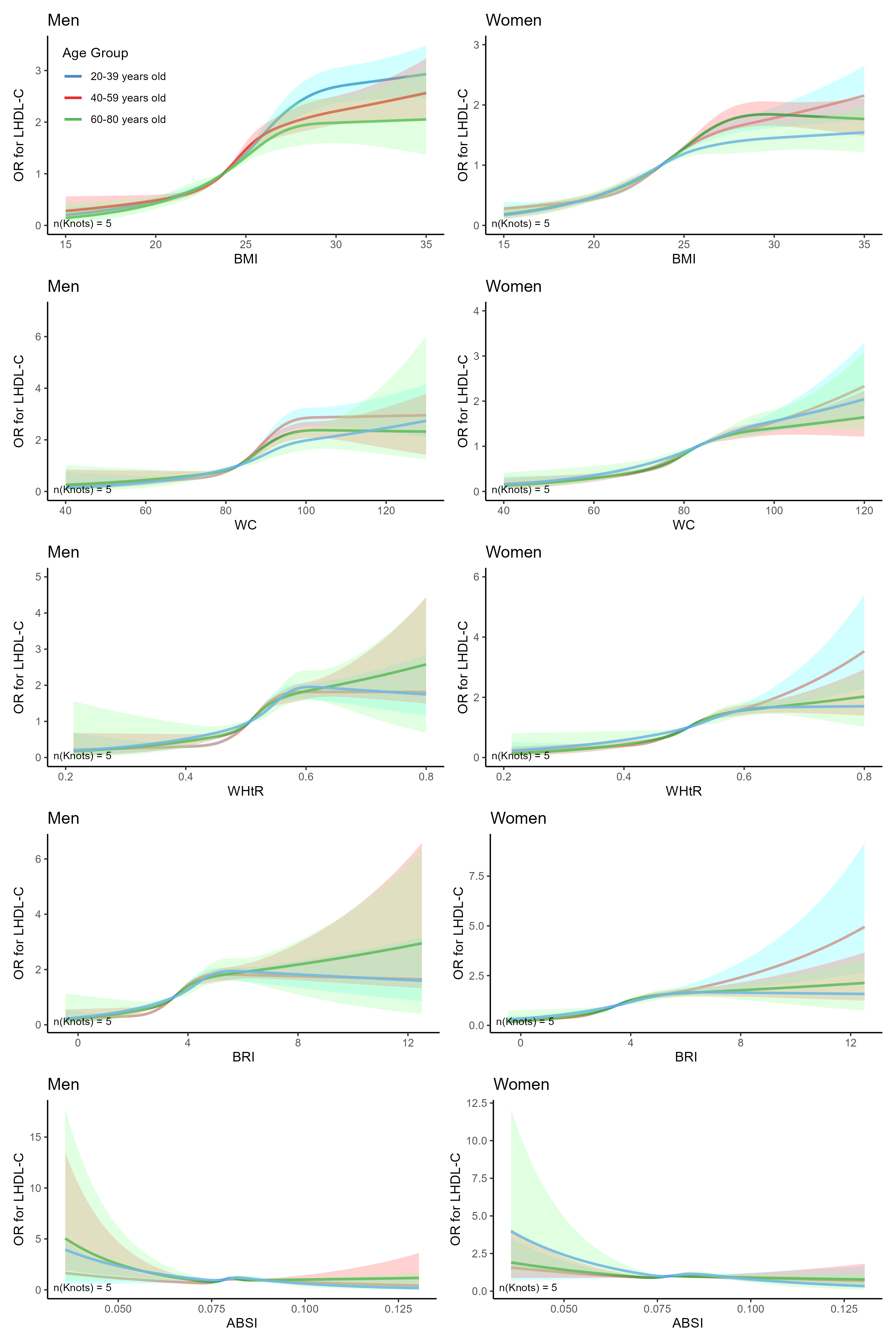

Supplement: Supplementary file 5 — Additional file 5: Figure S5. Restricted cubic splines representing the ORs for Low HDL-C status under different age stratifications in adjusted models. The relationship between the anthropometric indices and ORs for low HDL-C status. The regions of the three colours represent 95% CIs of the combined curve for that colour. [file 12889_2024_20505_MOESM5_ESM.tiff]
