# Supplementary material for: Associations between various anthropometric indices and hypertension and hyperlipidaemia: a cross-sectional study in China
Source: BMC Public Health. 2024 Nov 4;24:3045. doi: 10.1186/s12889-024-20505-w (PMC11536874; doi:10.1186/s12889-024-20505-w)
Supplement: Supplementary file 6 — Additional file 6: Table S1. Area under curve (AUC) of each anthropometric indices for hypertension and hyperlipidaemia in male and female genders in adjusted model II. [file 12889_2024_20505_MOESM6_ESM.docx]

**Additional file 6: Table S1. Area under curve (AUC) of each anthropometric indices for hypertension and hyperlipidaemia in male and female genders in adjusted model II.**

| **Anthropometric index** | **BMI** | | | **WC** | | | **WHtR** | | | **BRI** | | | **ABSI** | | |  |
| --- | --- | --- | --- | --- | --- | --- | --- | --- | --- | --- | --- | --- | --- | --- | --- | --- |
|  | **AUC** | **CI95%** | | **AUC** | **CI95%** | | **AUC** | **CI95%** | | **AUC** | **CI95%** | | **AUC** | **CI95%** | |  |
| **Hypertension** | | | | | | | | | | | | | | | |  |
| Total | 0.679 | | 0.674-0.683 | 0.692 | | 0.687-0.696 | 0.703^c^ | | 0.698-0.707 | 0.703^c^ | | 0.698-0.707 | 0.601 | | 0.596-0.605 | |
| Male | 0.658^a^ | | 0.652-0.664 | 0.659^a^ | | 0.652-0.665 | 0.679^c^ | | 0.672-0.685 | 0.679^c^ | | 0.672-0.685 | 0.574 | | 0.567-0.580 | |
| Female | 0.686 | | 0.679-0.692 | 0.703 | | 0.696-0.709 | 0.725^c^ | | 0.719-0.731 | 0.725^c^ | | 0.719-0.731 | 0.614 | | 0.607-0.621 | |
| **Hypertriglyceridaemia** | | | | | | | | | | | | | | | |  |
| Total | 0.706^a^ | 0.701-0.710 | | 0.710^a^ | 0.705-0.714 | | 0.697^c^ | 0.692-0.701 | | 0.697^c^ | 0.692-0.701 | | 0.578 | 0.573-0.583 | |  |
| Male | 0.704 | 0.697-0.709 | | 0.698 | 0.692-0.704 | | 0.696^c^ | 0.689-0.701 | | 0.696^c^ | 0.689-0.701 | | 0.562 | 0.555-0.568 | |  |
| Female | 0.689^a^ | 0.682-0.695 | | 0.684^a^ | 0.677-0.690 | | 0.695^c^ | 0.687-0.701 | | 0.695^c^ | 0.687-0.701 | | 0.573 | 0.565-0.580 | |  |
| **Hypercholesterolaemia** | | | | | | | | | | | | | | | |  |
| Total | 0.602 | 0.596-0.606 | | 0.612 | 0.607-0.617 | | 0.636^c^ | 0.631-0.640 | | 0.636^c^ | 0.631-0.640 | | 0.582 | 0.577-0.586 | |  |
| Male | 0.608^a^ | 0.600-0.614 | | 0.615^a^ | 0.608-0.622 | | 0.633^c^ | 0.626-0.639 | | 0.633^c^ | 0.626-0.639 | | 0.573 | 0.565-0.579 | |  |
| Female | 0.598 | 0.590-0.604 | | 0.618 | 0.611-0.624 | | 0.638^c^ | 0.631-0.644 | | 0.638^c^ | 0.631-0.644 | | 0.590 | 0.582-0.596 | |  |
| **High LDL-C** | | | | | | | | | | | | | | | |  |
| Total | 0.637 | 0.632-0.642 | | 0.654 | 0.648-0.658 | | 0.662^c^ | 0.657-0.667 | | 0.662^c^ | 0.657-0.667 | | 0.593 | 0.588-0.598 | |  |
| Male | 0.633 | 0.625-0.640 | | 0.641^b^ | 0.633-0.647 | | 0.651^bc^ | 0.644-0.658 | | 0.651^bc^ | 0.644-0.658 | | 0.574 | 0.566-0.581 | |  |
| Female | 0.634 | 0.627-0.641 | | 0.612 | 0.604-0.618 | | 0.605^c^ | 0.598-0.612 | | 0.605^c^ | 0.598-0.612 | | 0.508 | 0.501-0.515 | |  |
| **Low HDL-C** | | | | | | | | | | | | | | | |  |
| Total | 0.624 | 0.618-0.629 | | 0.583 | 0.577-0.588 | | 0.609^c^ | 0.603-0.613 | | 0.609^c^ | 0.603-0.613 | | 0.499 | 0.493-0.504 | |  |
| Male | 0.664 | 0.655-0.671 | | 0.649 | 0.640-0.657 | | 0.639^c^ | 0.630-0.646 | | 0.639^c^ | 0.630-0.646 | | 0.522 | 0.512-0.530 | |  |
| Female | 0.634 | 0.627-0.641 | | 0.612 | 0.604-0.618 | | 0.605^c^ | 0.598-0.612 | | 0.605^c^ | 0.598-0.612 | | 0.508 | 0.501-0.515 | |  |

Groups that share the same superscript letter do not exhibit any statistical difference between them. Conversely, a superscript with no letter indicates that the group is statistically different from all other groups

Model II: For the overall population, adjustments were made for sex, age, education, income, smoking status, urban or rural residence, ethnicity, salt intake and diabetes. For sex-specific subgroups, the adjustments included age, education, income, smoking status, urban or rural residence, ethnicity, salt intake and diabetes.
